# Sedentary behavior and physical activity are longitudinally associated with diurnal cortisol rhythms in individuals up to 1 year after colorectal cancer treatment: A prospective cohort study

**DOI:** 10.21203/rs.3.rs-10268142/v1

**Published:** 2026-07-16

**Authors:** Koen G. Frenken, Frank A.J.L. Scheer, Jingyi Qian, Martijn J.L. Bours, Stéphanie O. Breukink, Maryska L.G. Janssen-Heijnen, Joop Konsten, Eric T.P. Keulen, Judith A.P. Bons, Kenneth Meijer, Karen Steindorf, Judith de Vos-Geelen, Matty P. Weijenberg, Laurien M. Buffart, Eline H. van Roekel

**Affiliations:** 1 Department of Epidemiology, GROW Research Institute for Oncology and Reproduction, Maastricht University, Maastricht, the Netherlands; 2 Division of Sleep Medicine, Harvard Medical School, Boston, MA, USA; 3 Medical Chronobiology Program, Division of Sleep and Circadian Disorders, Departments of Medicine and Neurology, Brigham and Women’s Hospital, Boston, MA, USA; 4 Department of Surgery, GROW Research Institute for Oncology and Reproduction, NUTRIM Research Institute of Nutrition and Translational Research in Metabolism, Maastricht University Medical Centre+, Maastricht, the Netherlands; 5 Department of Clinical Epidemiology, VieCuri Medical Centre, Venlo, the Netherlands; 6 Department of Surgery, VieCuri Medical Centre, Venlo, the Netherlands; 7 Department of Internal Medicine and Gastroenterology, Zuyderland Medical Centre Sittard-Geleen, Geleen, the Netherlands; 8 Department of Immunodiagnostics, Central Diagnostic Laboratory, Maastricht University Medical Center+, Maastricht, the Netherlands; 9 Department of Nutrition and Movement Sciences, NUTRIM Research Institute of Nutrition and Translational Research in Metabolism, Maastricht University, Maastricht, the Netherlands; 10 Division of Physical Activity, Cancer Prevention and Survivorship, German Cancer Research Center (DKFZ), Heidelberg, Germany; 11 Department of Internal Medicine, Division of Medical Oncology, GROW Research Institute for Oncology and Reproduction, Maastricht University Medical Centre, Maastricht, the Netherlands; 12 Department of Medical BioSciences, Radboud University Medical Center, Nijmegen, the Netherlands; 13 Exercise Medicine Research Institute, Edith Cowan University, Perth, Western Australia, Australia.

**Keywords:** Diurnal cortisol pattern, colorectal cancer survivorship, physical activity, sedentary behavior, circadian rhythm

## Abstract

**Background:**

Less robust diurnal cortisol rhythms are hypothesized to be associated with fatigue, depressive symptoms, psychological distress, sleep disturbances and poorer quality of life. Sedentary behavior and physical activity may influence cortisol rhythmicity, providing mechanistic leads for intervention strategies. We examined longitudinal associations of accelerometer-assessed sedentary behavior and physical activity with diurnal cortisol patterns among patients with colorectal cancer (CRC) up to 12 months post-treatment.

**Methods:**

Among 74 CRC survivors, total and prolonged sedentary time (in bouts ≥30 min), total physical activity (TPA), and relative amplitude of physical activity reflecting day-night contrast in activity were determined with 7-day thigh-worn accelerometry at 6 weeks, 6 months, and 12 months after cancer treatment. Salivary cortisol was sampled five times daily over two consecutive days at each follow-up timepoint. As a comprehensive assessment of diurnal cortisol rhythms, the relative amplitude of cortisol concentration (RA_cort), diurnal slope, area under the curve (AUC) and cortisol awakening response (CAR) were derived as comprehensive measures of diurnal cortisol rhythms. We used confounder-adjusted linear mixed models to estimate overall longitudinal associations, with coefficients (β) reflecting differences in SD outcome units.

**Results:**

More total sedentary time (per 2 hours/day) was associated with a weaker decline in cortisol over time and thus a flatter slope (β=0.19; 95%CI=0.06,0.33). More prolonged sedentary time (per 2 hours/day) was associated with a lower RA_cort (β=−0.12; 95%CI=0.23, −0.01) and flatter cortisol slope (β=0.16; 95%CI=0.04,0.27). More TPA (per 1 hour/day: β=0.23; 95%CI=0.03,0.43) and a higher relative amplitude of physical activity (per SD: β= 3.51; 95%CI=0.10,6.92) were associated with a higher RA_cort. More total sedentary time (per 2 hours/day) was associated with a higher CAR (β=0.14; 95%CI=0.01,0.27).

**Conclusion:**

Higher physical activity levels, a larger RA of physical activity, and less sedentary time were associated with more robust diurnal cortisol rhythms, suggesting these behaviors may be targets for interventions aimed at improving cortisol rhythmicity after CRC.

**Registration:**

EnCoRe study (https://www.onderzoekmetmensen.nl/).

## Background

Colorectal cancer (CRC) is the third most diagnosed cancer type, with rising global incidence driven by population ageing, unhealthy lifestyles including the adoption of westernized dietary patterns (1). Earlier detection through widespread screening programs in most developed countries and advancements in treatments have resulted in higher survival rates, with five-year survival rates range between 74–96% for stage I-III and 11% for stage IV (1, 2). Consequently, the number of CRC survivors is growing, many of whom experience long-term side effects of the cancer and its treatment. Fatigue is among the most common and distressing complaints and can persist for years after treatment completion (3–10). Other commonly reported symptoms include depression, psychological distress, sleep disturbances and poorer quality of life (3–10). These symptoms are hypothesized to be at least partly linked with less robust diurnal cortisol rhythms (3–10).

Cortisol secretion is regulated by the hypothalamic-pituitary-adrenal (HPA) axis (11). In healthy individuals, cortisol secretion follows a diurnal rhythm characterized by rising levels across the second half of the sleep episode, a peak shortly after awakening, and a gradual decline across the day known as the diurnal cortisol slope (12–14). Several additional markers are commonly used to characterize HPA axis functioning via cortisol parameters including the relative amplitude (RA_cort) capturing the magnitude of 24-h variation, serving as an index of circadian rhythm robustness (12, 14, 15), and the total daily cortisol production captured by the 24-hour area under the curve (AUC) (12, 14, 15). These measures exclude a non-diurnal morning response known as the cortisol awakening response (CAR) (12, 14, 15). The CAR is characterized by a temporary increase in cortisol peaking within the first 30–45 minutes after awakening, primarily triggered by the sleep-wake transition and factors such as the dark-light transition and anticipated daily demands and stress (12, 16–18). Alterations in diurnal rhythm cortisol parameters are considered indicators of circadian dysregulation and downstream HPA axis dysregulation and may represent a biological pathway contributing to fatigue-related conditions, including cancer-related fatigue, fibromyalgia, and depression (4, 5, 19–21). Identifying modifiable lifestyle factors that may improve cortisol rhythmicity is therefore of clinical interest.

Physical activity may help synchronize the circadian system by restoring biological alignment through both non-photic and photic pathways (22). Non-photic effects may occur via resetting of peripheral oscillators, whereas photic effects may arise indirectly during outdoor activity through increased daylight exposure, the strongest zeitgeber for the central circadian clock (22–25). In contrast, sedentary behavior was found to be associated with disruption in cortisol regulation and may be associated with inflammatory processes (26, 27). A recent meta-analysis in non-cancer populations reported that higher physical activity levels were associated with steeper daily cortisol slopes, yet evidence in cancer survivors remains limited since existing studies are often cross-sectional and rarely based on objective measures of physical activity (23, 28, 29). Understanding whether these modifiable behavioral factors are associated with cortisol rhythmicity during the first-year after CRC treatment could help identify targets for interventions aimed at improving cortisol rhythmicity and thereby potentially reduce symptoms like fatigue after CRC.

Therefore, we aimed to examine longitudinal associations of accelerometer-assessed sedentary behavior and physical activity with diurnal cortisol patterns among survivors of CRC for up to 12 months post-treatment. We hypothesized that less sedentary behavior, more physical activity, and more standing are longitudinally associated with an increased RA_cort, a steeper negative slope across the wake episode, lower AUC, and a lower CAR.

## Methods

### Study design and population

Data were collected as part of the Energy for Life after ColoRectal cancer (EnCoRe) study, a prospective cohort study of survivors of stage I-III CRC in three centers in the Netherlands. (Registered at https://www.onderzoekmetmensen.nl/en). Between April 2012 and May 2025, CRC survivors of 18 years or older with stage I-III CRC were enrolled. To be eligible, patients had to live in the Netherlands, speak and understand Dutch, and have no comorbidities that could hinder participation (i.e., visual impairments or cognitive disorders). In this sub study, we only included patients from whom saliva was collected. Saliva samples were collected only for post-treatment measurements starting from the 1^st^ of August 2022 until the 1^st^ of May 2025. All measurements were performed at participants’ homes at diagnosis, at 6 weeks, 6 months and 12 months post-treatment.

The EnCoRe study and the subsequent amendment to include saliva sample collection were approved by the Medical Ethics Committee of the University Hospital Maastricht and Maastricht University (METC 11–3-075). All participants provided written informed consent.

### Sedentary behavior and physical activity

Time spent in sedentary behavior, standing, and physical activity were assessed objectively at all post-treatment time points using the validated tri-axial MOX activity monitor (MMOXX1, upgraded version of the CAM; Maastricht Instruments B.V., Maastricht, The Netherlands) (30). The accelerometer was worn on the right anterior upper thigh for seven consecutive days (24 hours/day) at each post-treatment time point. The accelerometer recorded raw accelerations in three orthogonal sensor axes at a sampling rate of 25 Hz. Accelerometer data were processed using a custom MATLAB program (version R2022a; The MathWorks, Inc., Natick, MA). For these analyses, data on hours/day of total sedentary time and prolonged sedentary time (uninterrupted bouts of at least 30 minutes of sedentary behavior, i.e. sitting or lying, including potential naps, during waking hours at a low intensity of ≤ 1.5 METs), were used (31–34). Standing time was defined as time spent upright during self-reported waking hours with an energy expenditure of ≤1.5 METs (33, 35). Total physical activity was defined as any movement or posture during self-reported waking hours exceeding 1.5 METs (36). A measurement day was considered valid if it included at least 10 hours of valid wear time (37). All variables were calculated for each valid day (≥10 hours of wear time (37)) and averaged across all available days at each post-treatment time point. For this analysis, only data from post-treatment measurements with at least four valid days were included.

The MOX accelerometer was also utilized to determine parameters of RAR including the mesor and amplitude. These parameters were calculated as part of the custom-made MATLAB program that was used to process accelerometer data and calculated for every valid day and subsequently averaged across all days available at each post-treatment time point. The cosinor method, which is widely used in RAR studies (38–41). The midline estimating statistic of the rhythm (mesor) is the mean of the activity counts across the 24-hour day where higher values demonstrate more activity across the 24-hour day (42, 43). The amplitude is the difference between the highest activity peak of the cosinor curve and the mesor in activity counts. Higher values for amplitude indicate a larger contrast between average activity levels and peak activity (42–44). Relative amplitude of physical activity was determined objectively by dividing the amplitude of the physical activity rhythm by the MESOR (43). A higher relative amplitude of physical activity reflects a larger day-night contrast in activity.

The MOX accelerometer has demonstrated moderate to high reproducibility and high validity for measuring sedentary behavior, standing and total physical activity (45). Its reliability in differentiating between various intensity levels of physical activity (i.e. light-intensity physical activity (LPA) and moderate-to-vigorous physical activity (MVPA)) is limited, and therefore only total physical activity was determined with the MOX accelerometer (45). Therefore, the validated Short QUestionnaire to ASsess Health-enhancing physical activity (SQUASH) was used to determine self-reported time spent in LPA and MVPA (46). Participants reported time spent on commuting, household work, and leisure time activities over a week. Each activity was assigned a MET value using Ainsworth’s Compendium of Physical Activities (47). Total weekly LPA and MVPA (hours/week) were calculated by summing the time spent on activities with energy expenditures of 1.5–3.0 METs and >3.0 METs, respectively (36). This was subsequently divided by 7 to achieve hours per day of total weekly LPA and MVPA. The SQUASH has demonstrated moderate reliability (test–retest: Spearman’s ρ =0.57–0.58) (48), and a relative validity that is comparable to other physical activity questionnaires when compared to accelerometer data (Spearman’s ρ =0.40 for moderate-intensity activities) (48).

### Diurnal cortisol measurement

Saliva samples were collected in pre-labeled Salivettes (Sarstedt, Germany). Participants were instructed to collect saliva samples on two consecutive weekdays. Participants were provided with detailed written and verbal instructions. On both days, saliva samples were collected upon awakening (T1), 30 minutes after waking (T2), at noon (T3), at 5 pm (T4), and at 11 pm or just before bedtime if participants went to bed earlier than 11 pm (T5). Additionally, participants were instructed to not eat, drink and brush their teeth in the 30 minutes prior to saliva collection, as food residue in the oral cavity may change salivary pH or composition (49). Saliva samples were refrigerated at home and subsequently returned by mail using appropriate safety envelopes for biological material (50).

Once the samples arrived at the university, they were centrifuged and stored at −80 °C (ensuring stability during long-term storage (50)) until laboratory analysis. Measurements of salivary cortisol concentrations (nmol/l) were performed at the Central Diagnostic Laboratory of the Maastricht University Medical Center+ using liquid chromatography-tandem mass spectrometry (LC-MS/MS). Specifically, the ChromSystems-kit at the Waters Xevo TQ-XS LC-MS/MS was applied, with a detectable range of 0.5 – 500 nmol/l. Using two laboratory control samples of 2.5 nmol/L and 20 nmol/L, a mean inter-assay coefficient of variation (CV) of 9.0% and 4.9% respectively, were observed during the laboratory analysis. We excluded the 6-week measurement of one participant because the sum of the raw cortisol concentration over the two measured days was below 8 nmol/l, which was likely due to incorrect measurement of the salivary cortisol.

### Relative amplitude of the cortisol concentrations (RA_cort)

Diurnal cortisol amplitude was operationalized as the RA_cort, calculated as the difference between the daily maximum (C_max_) and minimum cortisol (C_min_) concentrations divided by the mean of the daytime cortisol measurements T1, T3, T4 and T5. The T2 measurement time point was excluded to avoid bias from the CAR, an acute post-awakening increase that may confound estimation of cortisol’s diurnal variation (20). Subsequently, this value was multiplied by 100.


$$\text{Relative}\;\text{Amplitude}=\frac{\text{Cmax}-\text{Cmin}}{\text{Mean}\;\text{cortisol}}*100$$

This metric reflects the magnitude of peak-to-trough variation controlled for the interindividual difference in mean cortisol levels, with higher values reflecting greater diurnal variation indicating a more robust diurnal cortisol rhythm.

### Diurnal slope

The diurnal slope was defined as the rate of decline in salivary cortisol concentrations across the waking day (12), and used as a proxy for the diurnal rhythmicity of cortisol. The diurnal slope was calculated for each participant using linear regression of the log-transformed cortisol values as dependent variable (T1, T3, T4 and T5; excluding T2 to avoid bias from the CAR), and time since awakening as the independent variable. The diurnal slope was subsequently converted into an average hourly change in log-transformed cortisol concentrations across the waking episode (time between T1 and T5, usually an approximately 16-hour period), whereby less negative (flatter) slopes indicate reduced diurnal decline and are indicative of circadian disruption. A positive regression coefficient reflects less negative slope, indicating a flatter slope closer to zero.

### Area under the cortisol concentration curve relative to the ground (AUC)

The AUC was determined to quantify total diurnal cortisol secretion. AUC was computed as the average AUC using trapezoidal estimation based on log-transformed cortisol concentrations of all timepoints (T1, T3, T4, T5), excluding T2 to avoid bias from the CAR (20, 51). Exact sampling times (in hours since awakening) were used in the trapezoidal estimation. The log-transformed cortisol concentrations of the AUC were subsequently converted per hour across the day (time between T1 and T5, usually an approximately 16-hour period). A higher AUC indicates an increased total cortisol output which can reflect chronic stress (12, 52).

### The Cortisol Awakening Response (CAR)

The CAR was defined as the increase in cortisol occurring within the first 30 minutes after awakening (53). Salivary cortisol was sampled immediately upon awakening (T1) and at 30 minutes post-awakening (T2). The log-transformed CAR was calculated as the percentage increase from awakening levels using the formula:

$$CAR\left(\%\right)=\frac{T2-T1}{T1}*100$$

Lower CAR values may reflect a blunted post-awakening response and may indicate altered HPA axis activity and health problems (54).

### Sociodemographic, lifestyle and clinical covariables

Sociodemographic characteristics, including age, sex and education level (low, medium or high; see Table 1 for details) were assessed at diagnosis by self-report. Smoking status (current, former or never) was self-reported at diagnosis and during each post-treatment measurement. Body mass index (BMI; kg/m^2^) was calculated at diagnosis and at post-treatment time points based on self-reported height (only at diagnosis) and weight (all measurement time points). BMI was categorized according to the categories defined by the World Health Organization (WHO): underweight (BMI < 18.5 kg/m^2^), normal weight (18.5 ≤ BMI < 25 kg/m^2^), overweight (25 ≤ BMI < 30 kg/m^2^), and obesity (BMI ≥ 30 kg/m^2^) (55). Comorbidities (0, 1 or ≥2) were assessed using the 13-item Comorbidity Questionnaire at each post-treatment time point (56). Clinical data, obtained from medical records, included cancer stage (I, II, or III), tumor site (colon, rectosigmoid, or rectum) and treatment type (surgery, neoadjuvant/adjuvant chemotherapy, and/or radiotherapy).

### Statistical analyses

Descriptive statistics were used to summarize sociodemographic, lifestyle, and clinical characteristics, as well as sedentary behavior and physical activity data and cortisol parameters at each post-treatment time point. Normally distributed quantitative variables were presented as mean ± SD, while non-normally distributed variables were presented as median with inter-quartile range (IQR). Categorical variables were described using frequencies and percentages.

Analyses were conducted using linear mixed effect models to account for the repeated and hierarchical structure of the data, in which two days of cortisol measurements were nested within each measurement point and multiple measurements over the one-year post-treatment period were nested within individuals. Models included random intercepts to account for individual variation in trajectories over time. Mixed effect models were used to determine longitudinal associations of sedentary behavior and physical activity variables (i.e. time spent in total sedentary behavior, prolonged sedentary behavior, standing and total physical activity, and the relative amplitude of physical activity based on the MOX, LPA and MVPA based on the SQUASH) with cortisol parameters (i.e. diurnal cortisol slope, CAR, the RA and the AUC) from 6 weeks to 12 months post-treatment. Total sedentary time and prolonged sedentary time were analyzed in 2-hour increments, while standing time, total physical activity, LPA and MVPA were analyzed in 1-hour increments per day, approximating the SD of these variables. The RA of physical activity was increased per unit change. For all cortisol parameters except the RA, cortisol concentrations were log-transformed, due to the skewed distribution of the outcomes and based on previous literature (4). Afterwards, all cortisol parameters were standardized resulting in regression coefficients of mixed models reflecting differences in SD units of these parameters.

To examine differences in cortisol profiles, participants were dichotomized into high and low groups using a median split for total sedentary time, total physical activity time, standing time and the relative amplitude of physical activity.

Longitudinal associations were adjusted for predetermined potential confounders including age, sex and cancer stage. In addition to overall longitudinal associations, intra- and interindividual associations were analyzed separately. Intra-individual associations (within-participant changes over time) were estimated by modeling individual deviations from the person-mean value while inter-individual associations (average differences between participants over time) were estimated by adding centered person mean values to the model (57).

All statistical analyses and figures were generated using R version 4.5.2 and R packages lme4, lmerTest, ggplot2, gtsummary, rstatix. Statistical significance was set at p < 0.05 (two-sided). Significance was set at p < 0.05 for interaction terms.

## Results

### Study population characteristics

**Figure 1.**
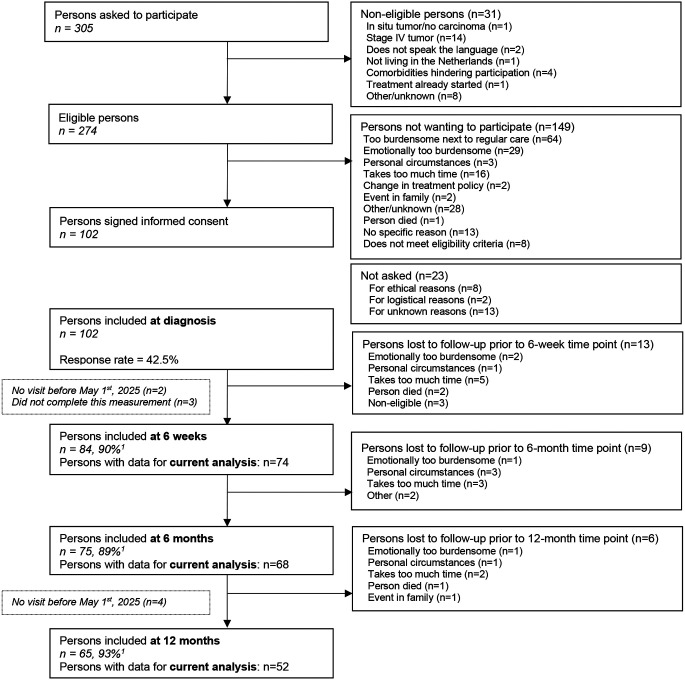
Flow diagram of the inclusion of participants within a substudy of the EnCoRe study and the number of post-treatment measurements included in the analyses during the time of cortisol exposure measurement. The exposure was collected during the visits from 1^st^ of August 2022 until 1^st^ of May 2025. ^1^Response rate = (persons with home visits)/ (persons with home visits+persons lost to follow-up – persons died). The declining number of participants at subsequent time points is partly since not all participants included had reached the 12-month time point in May 2025 (noted as no visit before may 1^st^, 2025).

A total of 81 survivors were included in the current analyses. For the separate time points, data were available from n=74 CRC survivors at 6 weeks post-treatment (Response rate: 91%, n = 68 at 6 months post-treatment (90%), and n=52 at 12 months post-treatment (93%). A flow diagram describing inclusion in the EnCoRe study and the number of measurements used in the current analyses can be found in Figure 1. At 6 weeks post-treatment, the average age was 66.3 (SD = 11.0) years and 52.7% of participants were male (Table 1). Most (78.4%) participants were medium-to-highly educated, 51.4% of participants never smoked while 1.4% were current smokers, and 60.8% were overweight or obesity. Most participants were diagnosed with colon cancer (87.8%), with the remaining being diagnosed with rectosigmoid or rectal cancer (12.2%). The predominant treatment was surgery (94.6%), while 36.5% received chemotherapy and 10.8% received radiotherapy. Of all patients, 23.0% received adjuvant CAPOX, 24% had a stage I diagnosis, while 38% received a stage II and III diagnosis. Just over half of the participants reported to have two or more comorbidities (54.1%).

### Descriptives of sedentary behavior, physical activity and cortisol parameters

**Figure 2.**
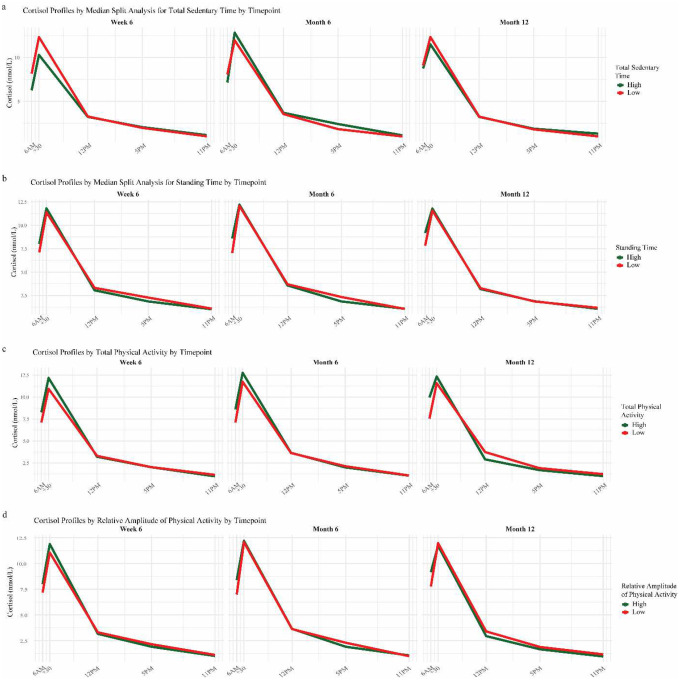
Differences in cortisol profiles across median-split behavioral groups. Panel A shows the high and low cortisol profiles for total sedentary time, Panel B shows the high and low cortisol profiles for standing time, Panel C shows the high and low cortisol profiles for total physical activity, and finally Panel D shows the high and low cortisol profiles for the relative amplitude of physical activity split by timepoint.

At 6 weeks post-treatment, participants spent on average 10.5 h/day (SD = 1.8) in total sedentary time, 4.0 h/day (SD = 2.4) in prolonged sedentary time, 3.2 h/day (SD = 1.2) standing, and 1.8 h/day (SD = 0.7) in total physical activity (Table 2). The median relative amplitude of physical activity based on MOX data was 0.1 (IQR: 0.1–0.2). The median self-reported LPA and MVPA were 1.4 h/day (IQR: 0.4–2.6) and 1.4 h/day (IQR: 0.6–2.1), respectively. Cortisol profiles split by high and low total sedentary time, total physical activity standing, and relative amplitude of physical activity show minimal differences between profiles in either high or low categories for all modifiable behaviors (Figure 2).

At 6 weeks post-treatment, the median RA_cort was 173.2% (IQR: 147.0, 214.0), the median diurnal cortisol slope was −1.85 × 10^−3^ nmol/l per hour (IQR: −2.45 × 10^−3^, −1.45 × 10^−3^), the median AUC was 4011 nmol/l (IQR: 3119, 4667), and the median CAR was 48.0% (IQR: 8.6, 121.0) (Table 2). The cortisol concentrations for each individual measurement time point over the day (averaged for the two consecutive days) are presented in Table 2 and visualized in supplementary figure 1. The diurnal profiles of mean cortisol concentrations showed a clear peak at 30 minutes after waking (signifying the CAR) followed by a clear decline towards the final cortisol collection point in the evening. Cortisol concentrations on day 1 and day 2 at the various time points showed similar profiles (Supplementary Figure 3). 10.7% at 6 weeks, 9.2% at 6 months, and 14.5% at 12 months post-treatment of total cortisol samples was missing due to an insufficient saliva volume sampled in the Salivette for the measurements.

### Longitudinal associations of sedentary behavior and physical activity with diurnal cortisol rhythms

**Figure 3.**
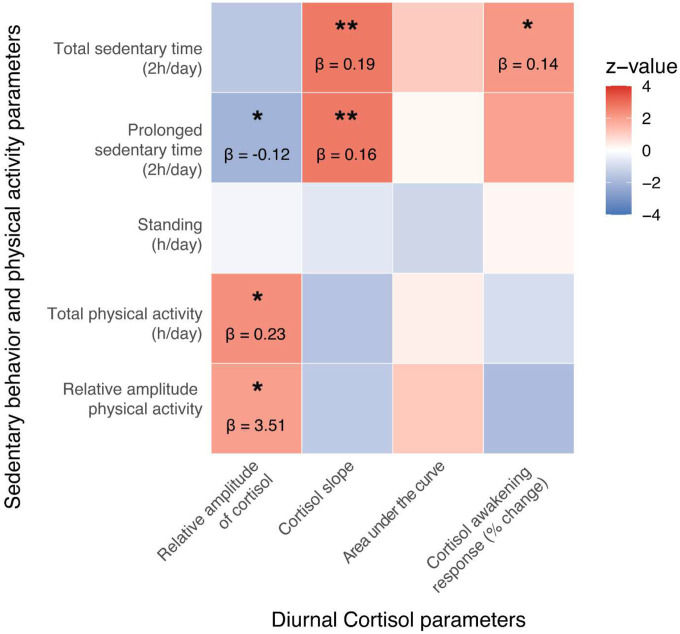
Heatmap that shows the direction and strength of overall longitudinal associations between objective measures of sedentary behavior and physical activity variables and diurnal cortisol parameters. One star indicates significance of p<0.05 and two stars indicate p<0.01. The colors represent Wald z-values (β/SE), reflect the strength and direction of the associations. The betas indicate difference over time in standardized outcome scores (SD units) per unit increase in exposure variables over time (e.g., per hour total physical activity,, see column labels). Beta coefficients of all longitudinal associations with 95% CIs, including tested inter- and intra-individual associations are depicted in Table 3.

More total sedentary time (per 2 hours/day) was associated with a weaker decline in cortisol over time, and thus with a flatter cortisol slope (β=0.19; 95%CI= 0.06, 0.33) (Figure 3 and Table 3). Associations were driven by both intra- and inter-individual associations (β=0.21; 95%CI= −0.00, 0.42 and β=0.18; 95%CI= 0.00, 0.36, respectively. More time spent in prolonged sedentary time (per 2 hours) was associated with a lower RA_cort (β=−0.12; 95%CI= −0.23, −0.01), and a flatter cortisol slope (β=0.16; 95%CI= 0.04, 0.27. Both associations were driven by inter-individual differences (β=−0.16; 95%CI= −0.30, −0.02 for RA_cort and β=0.17; 95%CI= 0.03, 0.31 for the slope). More total physical activity (per hour) was associated with a higher RA_cort (β=0.23;95%CI= 0.03, 0.43) over time, which was driven by inter-individual associations (β=0.29; 95%CI= 0.05, 0.52). A higher RA of physical activity was associated with a higher RA_cort (β=3.51; 95%CI= 0.10, 6.92), driven by inter-individual associations (β=4.62; 95%CI= 0.58, 8.66). A higher level of self-reported LPA was associated with a higher AUC (β=0.06; 95%CI= 0.01, 0.11), which was driven by intra-individual associations (β=0.08; 95%CI= 0.01, 0.14). Lastly, more total sedentary time (per 2 hours/day) was associated with a higher CAR (β=0.14; 95%CI= 0.01, 0.27) and this association was mainly driven by inter-individual associations (β=0.18; 95%CI= 0.02, 0.33). More prolonged sedentary time (per 2 hours/day) was associated with a higher inter-individual CAR (β=0.13; 95%CI= 0.01, 0.26). More standing (per 1 hour/day), was associated with a higher intra-individual CAR (β=0.35; 95%CI= 0.15, 0.55). A higher relative amplitude of physical activity (per SD) was associated with an inter-individual decrease in the CAR (β=−4.24; 95%CI= −7.66, −0.81).

## Discussion

This study examined longitudinal associations of sedentary behavior and physical activity variables with diurnal cortisol parameters in CRC survivors from 6 weeks to 12 months post-treatment. We found that more objectively assessed total sedentary time was associated with a flatter diurnal slope and with a higher CAR. Additionally, more prolonged sedentary time (in bouts with duration ≥30 min) was associated with a flatter diurnal slope and a lower RA_cort indicating a less robust rhythm. Additionally, more total physical activity time and a higher relative amplitude of physical activity, also assessed through accelerometry, were associated with a higher RA of the cortisol rhythm. Further, more self-reported LPA was longitudinally associated with a higher AUC. Overall, most associations were driven by inter-individual associations, but also intra-individual associations were observed indicating that within-person changes over time in physical activity and sedentary behavior were associated with cortisol rhythms. These findings indicate that more time spent sedentary is associated with a more disrupted cortisol rhythm, while more physical activity is associated with a more robust rhythm.

In line with our hypothesis, more total sedentary time was associated with a lower RA of cortisol and a flatter diurnal cortisol slope, reflecting a reduced contrast between the morning peak and nighttime nadir cortisol levels and this may be driven by poorer circadian regulation of the HPA axis and the autonomic nervous system which are responsible for the regulating of cortisol secretion in a diurnal pattern (14, 16). A blunted cortisol rhythm can indicate circadian disruption as cortisol is an established circadian phase marker (14, 58). Given that normal cortisol rhythmicity supports metabolic and energetic regulation, blunted patterns may therefore be indicative of diminished energy regulation affecting fatigue and causing low-grade inflammation (59, 60). Sedentary behavior is often accompanied by reduced exposure to natural daylight, a key zeitgeber for circadian entrainment (61, 62). Concurrently, greater exposure to artificial light and screen-based activities may suppress melatonin secretion and further altered circadian rhythm alignment (63).

We found that higher levels of physical activity, as well as a greater relative amplitude of physical activity, were associated with higher RA of cortisol, indicating a more pronounced contrast between cortisol’s morning peak and nighttime trough and therefore a more robust diurnal rhythm. Evidence linking physical activity with diurnal cortisol parameters after cancer remains limited. One prior study reported steeper diurnal cortisol slopes following a low-intensity yoga intervention in breast cancer survivors undergoing radiotherapy (64). A previous meta-analysis on various clinical and non-clinical populations has reported higher physical activity levels to be associated with steeper daily cortisol slopes (28). Mechanistically, regular physical activity may help synchronize the circadian system through both non-photic and photic pathways. Non-photic effects may occur via resetting of peripheral oscillators, whereas photic effects may arise through exposure to daylight, the strongest zeitgeber (22–25). Because circadian misalignment has been shown to increase inflammation (65, 66), improved circadian alignment through greater physical activity may help reduce pro-inflammatory markers. Indeed, more physical activity and reduced sedentary behavior have consistently been associated with lower levels of systemic inflammatory markers, including CRP, leptin and IL-6 in general populations (67, 68), with similar effects observed in breast cancer populations (69). In support of this, studies in patients with breast cancer and ovarian cancer have reported associations between blunted cortisol rhythms and negatively altered immune cell profiles (9, 69). Consequently, physical activity and sedentary behavior may influence cortisol rhythmicity through both circadian and inflammatory pathways (27).

Importantly, a well-defined diurnal cortisol rhythm, with a clear peak and trough, is a key marker for intact circadian regulation of the HPA axis and supports better energy regulation (59, 60), whereas blunted rhythms are implicated in negative health outcomes such as fatigue, depression and reduced quality of life (3–10). As prior studies have demonstrated associations between HPA axis dysregulation and increased fatigue burden, impaired cortisol rhythmicity may be a contributing mechanism (4, 20). In this context, our findings support the hypothesis that physical activity, through its various effects on circadian entrainment, alignment and inflammation, may improve health outcomes in cancer survivors.

There are some limitations within this study to consider when interpreting these findings. Some of the samples of cortisol could not be included in the data-analysis due to an insufficient saliva volume sampled in the Salivette per measurement (9.2–14.5% of measurements). However, mixed regression modelling techniques made optimal use of available data accounting for missing values, and we still found statistically significant findings. Related to this, based on the sample size available, we could only include the most important covariates in regression models, potentially leading to potential residual confounding from, for example medication use (e.g. corticosteroids), depression and sleep duration. Lastly, since this is an observational study, we cannot draw causal conclusions based on our findings.

Despite these limitations, there are also strengths within this study. Both physical activity and sedentary behavior as well as cortisol parameters were measured objectively, reducing potential information bias. In addition, the study design, which utilized repeated measures at three time points with five measurements per day, on two consecutive days, allowed incorporation of day-to-day differences as well as longitudinal investigation via linear mixed models up to 12 months after treatment, which is unique in this population.

## Conclusion

Our findings indicate that in the first year after CRC treatment, more physical activity was associated with improved rhythmicity in diurnal cortisol parameters while more sedentary behavior was associated with disrupted rhythms. Future studies should test whether interventions aimed at increasing physical activity and decreasing sedentary behavior can improve cortisol rhythmicity and health outcomes after CRC.

## Supplementary Material

1

## Figures and Tables

**Table 1.** Sociodemographic, lifestyle, and clinical characteristics in the study population of survivors of colorectal cancer.

Characteristics	6 weeks post-treatment (*n* = 74)^1^	6 months post-treatment (n = 68)^1^	12 months post-treatment (n = 52)^1^
**Age (years), mean ± SD**	66.3 ± 11.0	67.2 ± 10.9	67.8 ± 10.9
**Sex (male), *n* (%)**	39 (52.7)	33 (48.5)	23 (44.2)
**Education level, *n* (%)** ^2^			
Low	16 (21.6)	15 (22.1)	12 (23.1)
Medium	25 (33.8)	24 (35.3)	18 (34.6)
High	33 (44.6)	29 (42.6)	22 (42.3)
**BMI (kg/m** ^**2**^ **), mean ± SD**	27.0 ± 5.0	27.3 ± 5.1	27.1 ± 5.2
**BMI categorical, *n* (%)**			
Underweight (<18.5 kg/m^2^)	0 (0.0)	0 (0.0)	0 (0.0)
Healthy weight (18.5 – 24.9 kg/m^2^)	29 (39.2)	26 (38.2)	21 (40.4)
Overweight (25.0 – 29.9 kg/m^2^)	28 (37.8)	24 (35.3)	18 (34.6)
Obese (≥ 30 kg/m^2^)	17 (23.0)	18 (26.5)	13 (25.0)
**Smoking status, *n* (%)**			
Never	38 (51.4)	36 (52.9)	26 (50.0)
Former	35 (47.3)	31 (45.6)	26 (50.0)
Current	1 (1.4)	1 (1.5)	0 (0.0)
**Treatment, *n* (%)**			
Surgery (yes)	70 (94.6)	65 (95.6)	49 (94.2)
Chemotherapy (yes)	27 (36.5)	24 (35.3)	16 (30.8)
Radiotherapy (yes)	8 (10.8)	6 (8.8)	5 (9.6)
**Tumor site, *n* (%)**			
Colon	65 (87.8)	61 (89.7)	46 (88.5)
Rectosigmoid & rectum	9 (12.2)	7 (10.3)	6 (11.5)
**Cancer stage, *n* (%)**			
Stage I	18 (24.3)	17 (25.0)	15 (28.8)
Stage II	28 (37.8)	26 (38.2)	21 (40.4)
Stage III	28 (37.8)	25 (36.8)	16 (30.8)
**Comorbidities, *n* (%)**			
0	17 (23.0)	16 (23.5)	15 (28.8)
1	17 (23.0)	17 (25.0)	13 (25.0)
≥2	40 (54.1)	35 (51.5)	24 (46.2)

Abbreviations: BMI, body mass index; SD, standard deviation.

1 Percentages may not add up to 100% due to rounding.

2 Highest attained education, low: no education, primary education, or basic vocational education; medium: advanced vocational education or senior secondary vocational education; high: senior secondary general education, higher professional education, or academic higher education.

**Table 2.** Descriptive analyses of sedentary behavior, physical activity and cortisol variables in the study population of survivors of colorectal cancer.

Characteristics	6 weeks post-treatment (n = 74)	6 months post-treatment (n = 68)^1^	12 months post-treatment (n = 52)^1^
**Sedentary behavior and physical activity variables**			
Total sedentary time (h/day), mean (± SD)	10.5 ± 1.8	10.6 ± 2.2	10.3 ± 1.9
Prolonged sedentary time (h/day), mean (± SD)	4.0 ± 2.4	4.5 ± 2.8	4.1 ± 2.3
Standing (h/day), mean (± SD)	3.2 ± 1.2	3.3 ± 1.3	3.3 ± 1.5
Total physical activity (h/day), mean (± SD)	1.8 ± 0.7	1.7 ± 0.7	1.7 ± 0.7
Relative amplitude of physical activity, median (IRQ)	0.1 (0.1 – 0.2)	0.2 (0.1 – 0.2)	0.2 (0.1 – 0.2)
Light-intensity physical activity (h/day), median (IRQ)	1.4 (0.4 – 2.6)	1.9 (0.6 – 4.7)	2.1 (0.5 – 3.8)
Moderate-to-vigorous intensity physical activity (h/day), median (IRQ)	1.4 (0.6 – 2.1)	1.3 (0.6 – 2.3)	1.7 (0.6 – 2.5)
**Cortisol parameters**			
Relative amplitude of cortisol, %, median (IQR)	173.2 (147.0, 214.0)	194.0 (155.9, 227.8)	195.0 (158.1, 225.5)
Diurnal cortisol slope, (nmol/l per hour), median (IQR)	−1.85 × 10^−3^ (−2.45 × 10^−3^, −1.45 × 10^−3^)	−2.08 × 10^−3^ (−2.63 × 10^−3^, −1.54 × 10^−3^)	−1.99 × 10^−3^ (−2.61 × 10^−3^, −1.56 × 10^−3^)
T1 cortisol (nmol/l), median (IQR)^2^	7.8 (5.5, 10.0)	8.6 (5.9, 12.0)	8.6 (6.8, 12.2)
T2 cortisol (nmol/l), median (IQR)^3^	11.8 (9.4, 14.8)	12.41 (9.8, 16.6)	12.2 (6.6, 16.1)
T3 cortisol (nmol/l), median (IQR)^4^	3.4 (2.7, 4.6)	3.57 (2.6, 4.8)	3.2 (2.6, 4.3)
T4 cortisol (nmol/l), median (IQR)	2.3 (1.5, 3.1)	2.20 (1.7, 2.9)	1.9 (1.4, 3.0)
T5 cortisol (nmol/l), median (IQR)	1.1 (0.8, 1.6)	1.13 (0.8, 1.6)	1.2 (0.9, 1.7)
Area under the curve relative to the ground, (nmol/l), median (IQR)	4011 (3119, 4667)	4029 (3289, 5032)	3875 (2969, 5136)
Cortisol awakening response % change, median (IQR)^5^	48.0 (8.6, 121.0)	54.8 (−7.5, 104.3)	36.0 (−0.4, 87.3)

Abbreviations: SD, standard deviation; IQR, interquartile range.

1 The decreasing absolute numbers are largely due to participants not having reached all post-treatment follow-up time points at the time of data acquisition in May 2025.

2 For the variables measured by the MOX including total sedentary time, prolonged sedentary time, standing, total physical activity and the relative amplitude of physical activity; n = 8 were missing at 6 weeks, n = 6 were missing at 6 months and n = 2 were missing at 12 months.

3 For T1, there were n=1 missing at 6 weeks and 12 months, for T2 there was n=1 missing at 12 months, for T3 there was n=1 missing at 12 months.^5^ Missing n=1 at 12 months

5 For the fatigue questionnaires, missing’s included n=3 at 6 weeks for the CIS total fatigue, n=3 at 6 weeks for the CIS activity related fatigue,

**Table 3.** Longitudinal associations of sedentary behavior and physical activity with cortisol parameters between 6 weeks and 12 months post-treatment in the study population of survivors of colorectal cancer.

		Relative amplitude of cortisol (per 1 SD)^5^		Diurnal slope (per 1 SD)^5^		Area under the curve (per 1 SD)^5^		Cortisol awakening response (per 1 SD)^5^	
		β^1^	95% CI	β^1^	95% CI	β^1^	95% CI	β^1^	95% CI
**Total sedentary time (2h/day)** *(MOX)*	Overall^1,2^	−0.10	(−0.23, 0.03)	**0.19**	**(0.06, 0.33)**	0.07	(−0.06, 0.21)	**0.14**	**(0.01, 0.27)**
	Intra^2,3^	−0.05	(−0.24, 0.15)	0.21	(−0.00, 0.42)	0.16	(−0.04, 0.35)	0.05	(−0.20, 0.29)
	Inter^2,4^	−0.15	(−0.32, 0.03)	**0.18**	**(0.00, 0.36)**	−0.00	(−0.19, 0.18)	**0.18**	**(0.02, 0.33)**
**Prolonged sedentary time (2h/day)** *(MOX)*	Overall^1,2^	**−0.12**	**(−0.23, −0.01)**	**0.16**	**(0.04, 0.27)**	0.01	(−0.10, 0.12)	0.10	(−0.00, 0.21)
	Intra^2,3^	−0.06	(−0.23, 0.11)	0.13	(−0.05, 0.30)	0.10	(−0.06, 0.26)	0.02	(−0.18, 0.22)
	Inter^2,4^	**−0.16**	**(−0.30, −0.02)**	**0.17**	**(0.03, 0.31)**	−0.06	(−0.21, 0.09)	**0.13**	**(0.01, 0.26)**
**Standing behavior (h/day)** *(MOX)*	Overall^1,2^	−0.02	(−0.12, 0.09)	−0.04	(−0.14, 0.07)	−0.06	(−0.16, 0.04)	0.01	(−0.09, 0.11)
	Intra^2,3^	−0.05	(−0.21, 0.11)	−0.05	(−0.21, 0.11)	−0.06	(−0.21, 0.08)	**0.35**	**(0.15, 0.55)**
	Inter^2,4^	0.01	(−0.13, 0.15)	−0.03	(−0.16, 0.11)	−0.05	(−0.19, 0.09)	−0.10	(−0.22, 0.01)
**Total physical activity (h/day)** *(MOX)*	Overall^1,2^	**0.23**	**(0.03, 0.43)**	−0.17	(−0.37, 0.03)	0.04	(−0.16, 0.24)	−0.09	(−0.28, 0.10)
	Intra^2,3^	0.11	(−0.24, 0.47)	−0.14	(−0.50, 0.22)	−0.05	(−0.38, 0.28)	0.21	(−0.22, 0.64)
	Inter^2,4^	**0.29**	**(0.05, 0.52)**	−0.18	(−0.43, 0.06)	0.09	(−0.16, 0.34)	−0.16	(−0.37, 0.04)
**Relative amplitude of physical activity** *(MOX)*	Overall^1,2^	**3.51**	**(0.10, 6.92)**	−2.58	(−6.08, 0.92)	1.99	(−1.48, 5.46)	−2.99	(−6.16, 0.19)
	Intra^2,3^	0.80	(−5.53, 7.14)	−3.18	(−9.62, 3.26)	1.25	(−4.75, 7.25)	3.75	(−4.15, 11.66)
	Inter^2,4^	**4.62**	**(0.58, 8.66)**	−2.33	(−6.50, 1.85)	2.37	(−1.89, 6.63)	**−4.24**	**(−7.66, −0.81)**
**Light-intensity physical activity (h/day)** *(SQUASH)*	Overall^1,2^	0.01	(−0.05, 0.06)	−0.01	(−0.07, 0.04)	**0.06**	**(0.01, 0.11)**	0.01	(−0.05, 0.06)
	Intra^2,3^	0.03	(−0.04, 0.10)	−0.04	(−0.11, 0.03)	**0.08**	**(0.01, 0.14)**	0.00	(−0.08, 0.08)
	Inter^2,4^	−0.03	(−0.11, 0.06)	0.03	(−0.06, 0.11)	0.03	(−0.05, 0.12)	0.00	(−0.07, 0.08)
**Moderate-to-vigorous-intensity physical activity (h/day)** *(SQUASH)*	Overall^1,2^	0.05	(−0.04, 0.13)	0.00	(−0.08, 0.09	0.07	(−0.01, 0.15)	−0.05	(−0.13, 0.04)
	Intra^2,3^	0.01	(−0.11, 0.14)	0.04	(−0.08, 0.17)	0.07	(−0.04, 0.18)	−0.00	(−0.14, 0.14)
	Inter^2,4^	0.08	(−0.04, 0.20)	−0.03	(−0.15, 0.09)	0.07	(−0.05, 0.20)	−0.07	(−0.17, 0.03)

Values in bold are statistically significant (P < 0.05).

1 The β-coefficients indicate the overall longitudinal difference in standardized outcome scores (SD units) per unit increase in the exposure variables over (e.g., per hour total physical activity).

2 Linear mixed-models were adjusted for sex (male/female), age at enrolment (years), clinical stage (I, II, III).

3 The β-coefficients indicate the average change in the outcome score over time when sedentary and physical activity parameters increase with 1–2 hours between time points from 6 weeks to 12 months post-treatment within individuals.

4 The β-coefficients indicate the average difference in the outcome score between individuals when sedentary and physical activity parameters increase with 1–2 hours between time points from 6 weeks to 12 months post-treatment.

5 Per SD unit for the relative amplitude is 1.96%, for the slope is 0.05 nmol/l per hour, for the area under the curve is 6.07 nmol/l, and for the relative amplitude is 64.14%.

Abbreviations: SQUASH: Short Questionnaire to Assess Health-enhancing physical activity

## Data Availability

Data described in the manuscript, code book, and analytic code will be made available upon request pending (e.g., application and approval, payment, other). Requests for data of the EnCoRe study can be sent to Dr. Martijn Bours, Department of Epidemiology, GROW Research Institute for Oncology and Reproduction, Maastricht University, the Netherlands (email: m.bours@maastrichtuniversity.nl).
