# Supplementary material for: Sedentary behavior and physical activity are longitudinally associated with diurnal cortisol rhythms in individuals up to 1 year after colorectal cancer treatment: A prospective cohort study
Source: Res Sq. 2026 Jul 16:rs.3.rs-10268142. Preprint. [Version 1] doi: 10.21203/rs.3.rs-10268142/v1 (PMC13405483; doi:10.21203/rs.3.rs-10268142/v1)
Supplement: 1 [file NIHPPRS10268142V1-supplement-1.pdf]

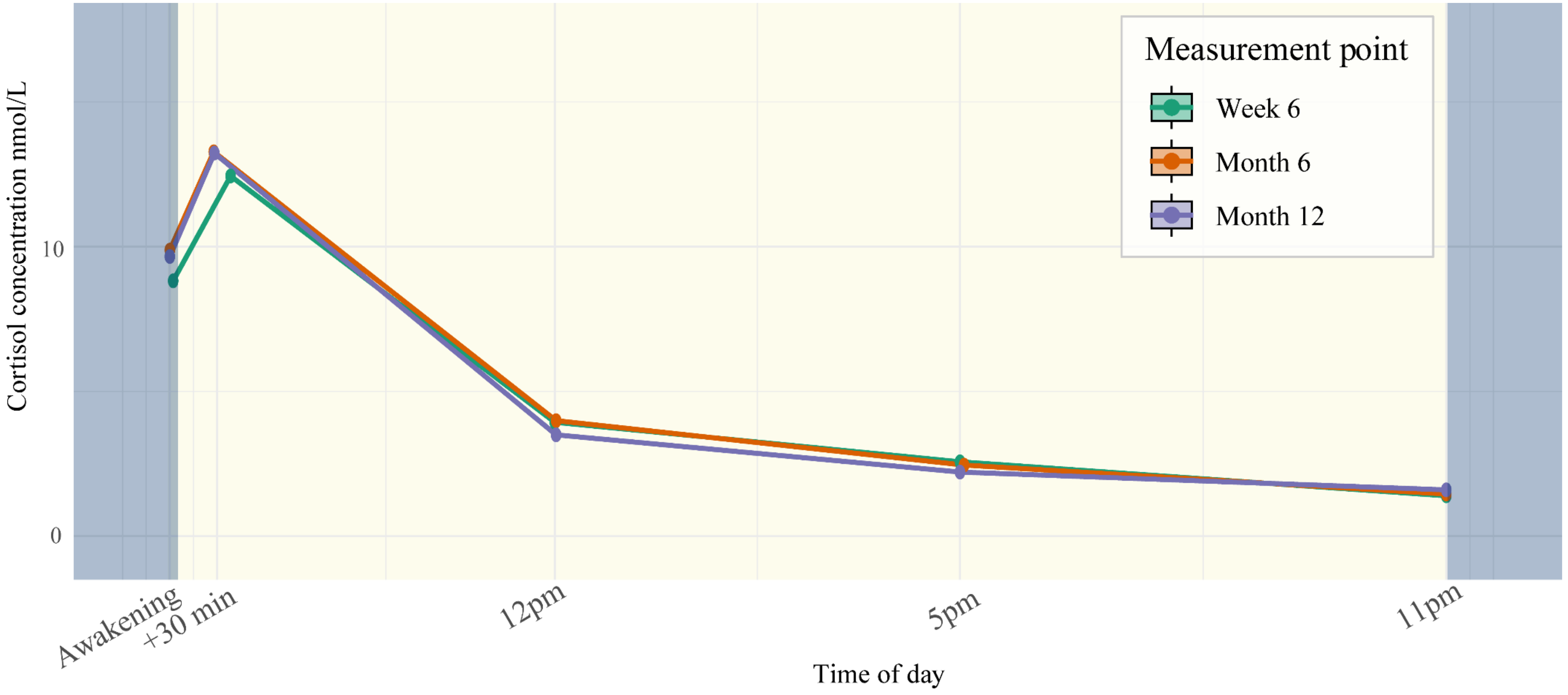


**Supplementary Figure 1**. Cortisol profiles of included survivors of CRC showing median cortisol concentrations at all 5 measurement points during the day (averaged over two-days) (x-axis), with colors representing the different measurement time points after the end of CRC treatment (see legend).

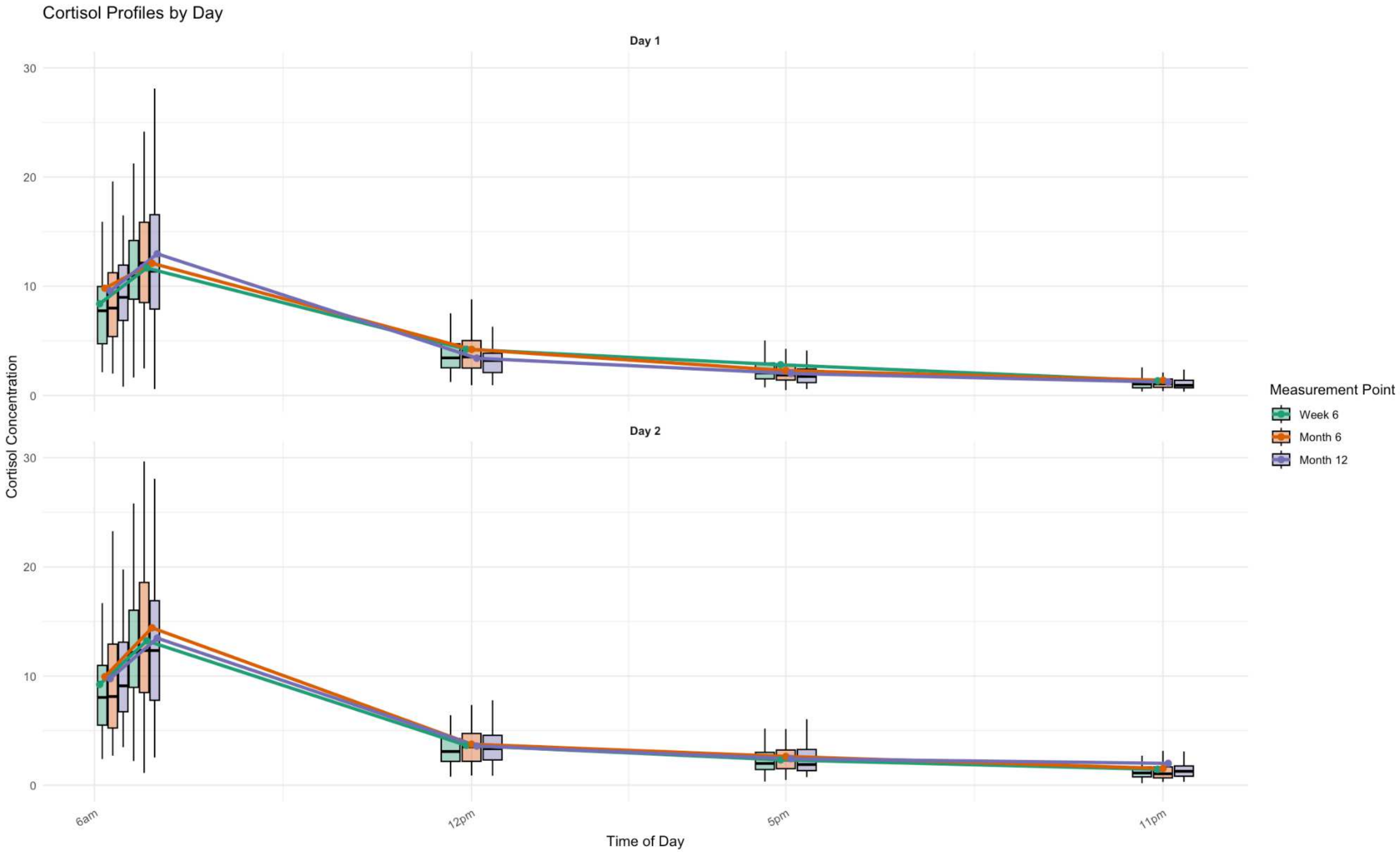


**Supplementary Figure 2**. Median cortisol profiles of EnCoRe population at all various measurement time points separated by day 1 and day 2.
